# Matrix Metalloproteinases 2 and 9 Immunoexpression in Prostate Carcinoma at the Positive Margin of Radical Prostatectomy Specimens

**DOI:** 10.1155/2014/262195

**Published:** 2014-07-06

**Authors:** Romano Oguić, Vladimir Mozetič, Eleonora Cini Tešar, Dora Fučkar Čupić, Elvira Mustać, Gordana Đorđević

**Affiliations:** ^1^Department of Urology, Clinical Hospital Centre Rijeka, Krešimirova 42, 51000 Rijeka, Croatia; ^2^Medico Polyclinic, Agatićeva 8, 51000 Rijeka, Croatia; ^3^Department of Oncology, Clinical Hospital Centre Rijeka, Krešimirova 42, 51000 Rijeka, Croatia; ^4^Department of Pathology, School of Medicine, University of Rijeka, Braće Branchetta 20, 51000 Rijeka, Croatia

## Abstract

The aim of this study was to evaluate the expression of matrix metalloproteinase 2 (MMP-2) and matrix metalloproteinase 9 (MMP-9) in prostate cancer in the main tumor mass and tumor cells at the positive margin as well as the influence of these biomarkers on the biochemical recurrence of the disease in prostatectomy patients. Tissue microarrays of 120 archival prostate carcinoma samples were immunohistochemically evaluated for MMP-2 and MMP-9 expression and compared with clinicopathological parameters. Tumors with positive surgical margins showed significantly higher overall expression of MMP-9 versus tumors with negative resection margins (*P* = 0.0121). MMP-9 expression was significantly elevated in tumors from patients who had biochemical recurrence (*P* = 0.0207). In the group of patients with negative margins, MMP-9 expression above the cut-off value was significantly associated with recurrence (*P* = 0.0065). Multivariate analysis indicated that MMP-9 is a good predictor of biochemical recurrence (odds ratio = 10.29; *P* = 0.0052). Expression of MMP-2 in tumor cells was significantly higher at the positive margins than in the main tumor mass (*P* = 0.0301). The present results highlight the potential value of MMP-2 and MMP-9 expression for predicting the behavior of prostate tumors after prostatectomy with both positive and negative surgical margins.

## 1. Introduction

Extensive studies of the matrix metalloproteinases (MMPs) indicate that a large number of MMP family factors play active roles in carcinogenesis and metastasis [1–3]. Many studies have focused on the role of MMPs and their inhibitors in the prognoses of various types of malignancies. Most published data on MMP-2 and MMP-9 address the role of these proteins in encouraging the aggressiveness of cancers. Increased expression of MMP-2 correlates with low survival in patients with breast cancer [4–8], is associated with a 4.5-fold higher relative risk of mortality from skin melanoma, and predicts the risk of metastasis in uveal melanoma [9, 10]. Increased expression of MMP-2 is a sign of poor prognosis in cancer of the stomach and pancreas [11–13] as well as the prostate [14].

Kuvaja et al. concluded that low levels of serum pro-MMP-2 correlate with aggressive cancer behavior [15]. High MMP-2 expression in hematological malignancies suggests good prognosis; in contrast, high MMP-9 expression is a sign of poor disease outcome [16]. The function of MMP-9 is still controversial in solid tumors. Scorilas et al. proposed that MMP-9 immunoreactive protein can be a favorable sign for node-negative breast cancer [17]. On the other hand, investigations of high MMP-9 levels in plasma or serum samples showed that MMP-9 expression may be associated with increased risk of recurrence and poor prognosis [18, 19]. In prostate carcinoma, MMP-2 and MMP-9 are novel molecular biomarkers that reflect the invasive and metastatic potential of this type of carcinoma [20]. The standard biomarkers that significantly predict the clinical and biochemical recurrence of prostate cancer are preoperative serum prostate-specific antigen (PSA), pathological grade according to Gleason score, positive surgical margins (with and without extraprostatic extension), and capsular incision adding up the following factors as of prognostic significance: presence of perineural, angiolymphatic, seminal vesicle invasion and extraprostatic tissue invasion. However, the real prognostic importance of positive margins remains to be defined. Few studies have emphasized the behavior of the previously mentioned biomarkers on the positive edge of the resection in comparison to their behavior in the bulk tumor mass. Recently, Cao et al. addressed the issue of positive resection margins in radical prostatectomy and found that the Gleason score at the edge of the tumor resection is predictive of biochemical recurrence [21]. There are currently no studies on the predictive value of MMP expression on the edge of the resection in radical prostatectomy.

The aim of this study was to measure the expression levels of MMP-2 and MMP-9 in the main tumor mass and in tumor cells on the positive margin and to compare these expression levels with Gleason score and tumor size in patients treated by radical prostatectomy. We also examined the influence of these biomarkers on biochemical recurrence of the disease in prostatectomy patients.

## 2. Methods

### 2.1. Patients

During the period from 2001 to 2006, acinar adenocarcinoma was discovered by ultrasound-guided biopsy of the prostate in 793 patients at the Clinic of Urology, Clinical Hospital Center Rijeka, Croatia. In accordance with the criteria from the European Urological Association, candidates for radical prostatectomy had a Gleason score of 7 or less, PSA levels below 10 ng/mL, and clinically confirmed prostate cancer in stage T1c/T2c (normal preoperative computed tomography findings and pelvic bone scintigraphy) [22]. From 2001 to 2006, 120 patients who had undergone radical prostatectomy were chosen from medical documentation files and adequate clinical data were collected. Two groups of patients were formed: a group of 71 patients having tumors with negative surgical margins and a second group of 49 patients with tumors showing positive surgical margins. Tumor material, obtained from radical prostatectomy, was selected for the construction of tissue microarrays. Representative areas on the hematoxylin and eosin-stained sections were carefully selected and marked on the corresponding paraffin blocks. From each carcinoma, two tissue cores (1 mm in diameter) were obtained from the main tumor mass and two cores were taken from the tumor at the positive surgical margin; these samples were arrayed in a recipient paraffin block using MTA Booster OI Manual Tissue Arrayer (Alphalyse, Plaisir, France).

### 2.2. Immunohistochemistry

Immunohistochemical staining for MMP-2 (17B11 mouse monoclonal antibody, Vision Biosystems Novocastra, Newcastle, UK) and MMP-9 (17W2 mouse monoclonal antibody NCL-MMP-9-493, Vision Biosystems Novocastra) was carried out with an automated immunostainer (OptiMax Plus, BioGenex) employing a standard biotin-streptavidin method. Heat-induced pretreatment for antigen retrieval (slides were immersed in a 10 mM citrated buffer, pH 6.0, at 95°C for 5 min) was carried out prior to incubation with primary antibody; the Dako En/Vision+/HRP Kit was used to visualize MMP expression. Intestinal tissue of patients with inflammatory bowel disease (as recommended by the manufacturer) and the positive control for MMP-9 were normal liver tissues (as recommended by the manufacturer).

The expression level of each protein was determined with image analysis system ISSA 3.1 software (Zagreb, Croatia). Staining was evaluated as the percentage of cytoplasmic-positive tumor cells multiplied by the staining intensity; this value was expressed as a histoscore. Clinicopathological data obtained from patient medical records included age, tumor size, TNM stage, Gleason score, and margin status of the prostatectomy specimens (Table 1). We separately examined the immunoexpression of the MMPs relative to localization within the tumor mass (MMP expression on the positive surgical margins and in the bulk of the tumor mass).

## 3. Results

**Figure 1:**
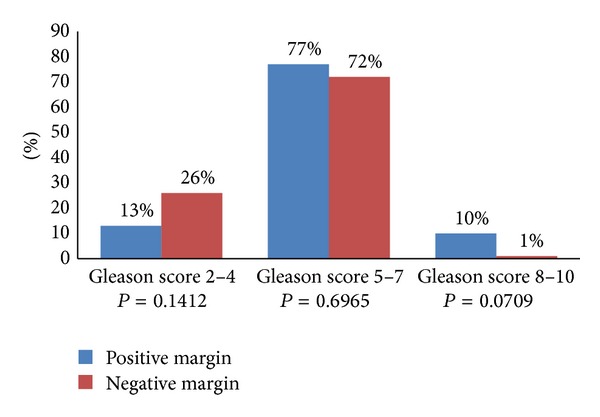
Analysis of Gleason score in patients with positive or negative surgical margins.

We found a mean Gleason score significantly higher in the group with positive surgical margins than in the group with negative surgical margins (*P* = 0.0032; Figure 1).

Immunohistochemical staining against MMP-2 and MMP-9 in glands with hyperplastic epithelium indicated weak or negative expression of these proteins, except for glands with high-grade PIN, where the expression was stronger (Figures 1). MMP-2 and MMP-9 immunoexpression in tumor cells was cytoplasmic, finely granular, and varied in intensity and percentage. We did not find tumors that were negative for the expression of these proteins (Figure 1).

MMP-2 was expressed not only in the cytoplasm of tumor cells but also in the cytoplasm of prostatic stromal cells, endothelium, fibroblasts, smooth muscle cells, macrophages, and lymphocytes. The average histoscore of MMP-2 expression per tumor was 98.9160 (standard deviation (SD) 50.1750). MMP-2 was significantly more strongly expressed in tumor cells on the positive margins than in the main tumor mass, with an average of 109.0237 (SD 64.0751; *P* = 0.0301). In the stroma, the strongest MMP-2 expression occurred in the cytoplasm of inflammatory cells, especially macrophages (Figure 3).

MMP-9 expression was detected in the cytoplasm of tumor cells, while stromal cells were weakly positive for this marker; staining could be categorized as cytoplasmic or nuclear plus cytoplasmic. The cytoplasm of some tumors contained more pronounced luminal expression of MMP-9, while, in other tumors, this staining was diffuse or mostly peripheral. The average amount of MMP-9 immunostaining was higher than that of MMP-2 in both examined locations. The mean MMP-9 expression was 142.1780 (SD 51.0358) in the main tumor mass and 171.0924 (SD 61.9203) at the positive margin of resection. Although there was no significant difference in MMP-9 immunoexpression between the main tumor mass and the edge, a group of tumors with positive surgical margins showed significantly stronger overall expression of MMP-9 compared to a group of tumors with negative resection margins (*P* = 0.0121; Table 2).

Increased expression of MMP-2 and MMP-9 was found in tumors of better differentiation and higher Gleason score but with no significant differences between tumors with Gleason score <7 and high-grade cancers with Gleason score ≥7. Regarding disease stage, MMP-2 staining was slightly stronger in higher-stage tumors but without statistical significance. Tumor size was significantly associated with MMP-9 expression, which was high in tumors larger than 1 cm (*P* = 0.038; Table 3).

**Figure 2:**
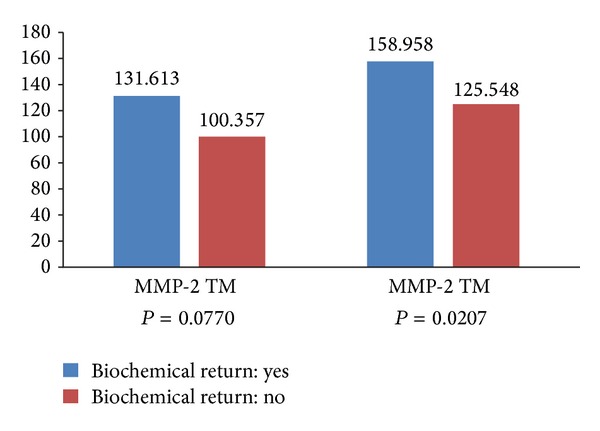
Biochemical recurrence with respect to molecular biomarkers in patients with tumors with negative surgical margins.

**Figure 3:**
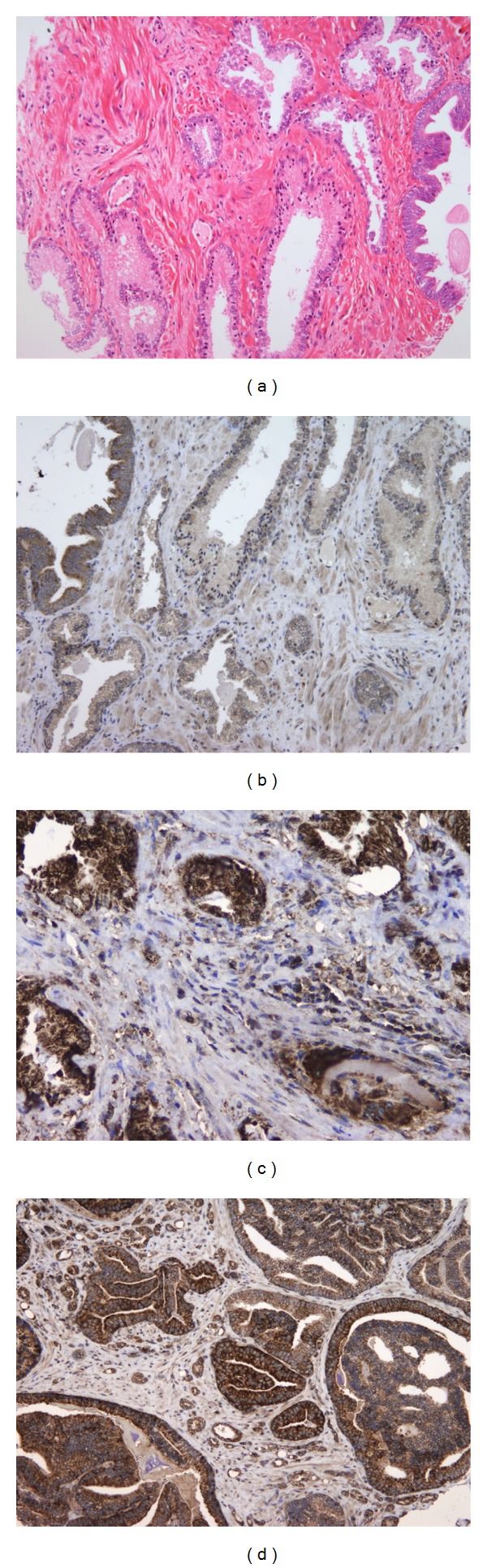
(a) Glands lined with hyperplastic epithelium and PIN (hematoxylin and eosin staining, 100x). (b) MMP-2 and MMP-9 immunohistochemistry indicates weak or negative expression in hyperplastic epithelium, except for the PIN, where the expression is strong (100x). (c) MMP-9 immunoexpression in moderately differentiated prostate cancer. Note the intense staining in the cytoplasm of tumor cells and the strongly positive reaction in the cytoplasm of stromal cells (200x). (d) MMP-2 immunoexpression in poorly differentiated prostate cancer. Note the strong cytoplasmic staining intensity (200x).

We analyzed the biochemical recurrence of the disease in relation to MMP expression in the group of patients with negative surgical margins that did not receive any therapy after prostatectomy that could have affected the outcome. MMP-9 expression was significantly elevated in tumors from patients who experienced biochemical recurrence (*P* = 0.0207). MMP-2 expression in tumors tended to be increased in patients with biochemical recurrence (*P* = 0.0770; Figure 2).

We used a receiver operating characteristic (ROC) analysis, which is widely recognized as a measure of a diagnostic test's discriminatory power. The maximum value for the area under the curve (AUC) is 1.0, indicating a (theoretically) perfect test (100% sensitive and 100% specific). An AUC value of 0.5 indicates no discriminative value. We evaluated MMP-2 and MMP-9 expression as predictors of recurrence in patients with tumors with negative surgical margins. MMP-9 expression was detected in all patients (Table 4). Furthermore, cut-off values for these parameters and groups were determined based on the Youden index (Table 5).

Analysis of the proportions of patients with and without recurrence in terms of increased MMP-9 expression revealed significant differences between the two groups at a significance level of 0.1 (*P* = 0.0635).

In the group of patients with negative margins, increased MMP-9 expression above the cut-off value of 175 was significantly associated with recurrence (*P* = 0.0065), while MMP-2 expression was not (Table 6).

Univariate and multivariate analysis showed that MMP-9 expression is a good predictor of biochemical recurrence (Table 7).

Recurrence-free survival was not significantly shorter in the group of patients with tumors with negative margins and high MMP-9 expression (Kaplan-Meier analysis, *P* = 0.5555).

## 4. Discussion

Prostate diseases are often present in the older population. Benign enlargement or inflammation of the prostate affect quality of life, while prostate cancer can be life threatening. Unfortunately, the routine diagnostic triad of a combination of an abnormal PSA level, digital rectal examination, and transrectal sonography-guided biopsy of the prostate does not always result in detection of the tumor (sensitivity 75%, specificity 68%). If one or more tests are abnormal, prostate biopsy must be performed to confirm or exclude malignant disease. For now, the best information about limitations, disease stage, and the malignant potential of the prostate cancer are obtained by histopathological examination.

Pathological classification of prostate cancer after radical prostatectomy provides important prognostic information, and an accurate histopathological report is thought to be fundamental for treating and monitoring patients. Preoperative serum PSA levels, pathological grade according to Gleason score, invasion into the seminal vesicle, positive surgical margins with and without extraprostatic extension, and capsular incision are significant predictors of clinical and biochemical recurrence. The real prognostic significance of positive margins and capsule incision remains to be defined [23, 24].

Approximately 5–43% of patients with radical prostatectomy have positive surgical margins, and only 25–47% of them develop biochemical recurrence [25]. These observations suggest the utility of further subclassification of positive margins in order to identify patients with higher risks of recurrence. In our study, the overall rate of positive surgical margins was 29.8% in pT3 tumors and up to 90% in other tumors, which is similar to other studies [26–28].

The time to recurrence, regardless of treatment, was significantly shorter in patients with locally confined tumors with positive surgical margins. This observation is consistent with the results of Bostwick et al., who reported a positive impact of positive prostatectomy margins on biochemical recurrence of the disease [29]. Few studies have emphasized the potential utilities of biomarker expression on positive surgical margins relative to the main tumor mass. In our study, which used tissue microarrays, the average Gleason score was significantly higher in the group of patients with tumors with positive margins, partially confirming the results of Cao et al. [30]. There was no significant difference between the average Gleason score on the margins of the tumor resection and the average Gleason score in the main tumor mass; this observation may be due to the strict selection of patients for prostatectomy, which usually requires Gleason scores ≤7.

The human family of MMPs consists of at least 24 proteinases involved in degradation of the extracellular matrix and components of the basement membrane. The MMPs are directly involved in the essential cellular processes of proliferation, differentiation, angiogenesis, and apoptosis through their ability to catalyze the hydrolysis of various substrates, including the precursors of cytokines, growth factors, and hormone receptors. A number of studies revealed positive correlations between tumor invasion and the activities of the gelatinases MMP-2 and MMP-9. Expression of these MMPs is most frequently associated with tumor aggressiveness and overall survival, and they are used as markers of malignant phenotypes [31–33].

In prostate cancer, MMP-2 and MMP-9 expression indicate the tumor's invasive and metastatic potential [34]. Given the development of specific MMP inhibitors, we assumed that the immunohistochemical determination of the expression of these two MMPs would be of particular importance for therapy, especially if it indicated whether the tumor was at high risk of recurrence. In our study, we sought to determine whether MMP-2 and/or MMP-9 expression differed between aggressive cancers on the positive margin of the resection that progress and cancers that remain localized. We detected a significant difference in MMP-2 expression on the positive margin versus that in the main tumor mass (*P* = 0.0301). MMP-9 expression at the margin of the resection did not significantly differ from the expression in the main tumor mass, but, in the group of tumors with positive margins, MMP-9 was significantly more strongly expressed overall versus the group of tumors with negative resection margins (*P* = 0.0121).

In the group of tumors with negative surgical margins, MMP-9 expression was significantly higher in samples from patients with biochemical recurrence of the disease. Therefore, this study yielded additional insight into the potential value of the biomarkers investigated here, particularly, the utility of MMP-2 and MMP-9 expression for predicting the invasive behavior of the tumor and justification for therapy. This utility has been highlighted by numerous works that have explored the expression of MMP-2 and MMP-9 in serum, urine, and tissues from prostate cancers. Sauer and colleagues linked higher expression of MMP-2 and MMP-9 in serum and tissues with higher Gleason scores [34]. In our study, MMP-2 and MMP-9 were expressed in tumors with poor differentiation and higher Gleason scores, but there were no significant differences between low Gleason score (<7) and high-grade cancer (Gleason score >7). These enzymes also displayed significantly higher values in the serum of metastatic carcinoma. In patients with pT3 tumors, increased expression of MMP-2 and MMP-9 was associated with shorter disease-free survival periods; the expression levels of these markers exhibited statistical significance in predicting recurrence. Significant decreases in the levels of these two markers in the sera of patients with metastatic disease after therapy are useful as independent predictors of disease stage in combination with the expression of adhesion molecules [35].

## 5. Conclusion

The results of this study underscore the potential value of the expression levels of MMP-2 and MMP-9 for predicting the invasive behavior of tumors with positive surgical margins; further investigation of this observation and its utility is warranted. In our patients with tumors with negative surgical margins, increased MMP-9 expression above the cut-off value was a good predictor of biochemical recurrence, whereas MMP-2 expression was associated with an increased risk of relapse.

## Figures and Tables

**Table 1:** Clinical and pathological features of patients with prostate cancer treated with prostatectomy.

Feature	
Age of patients (years, median)	64.12
Preoperative serum PSA (ng/mL, median)	8.50
Tumor size (cm, median)	2.25
Pathologic stage (%)	
pT2	74.00
pT3	26.00
Gleason score (%)	
≤6	65.40
≥7	34.60
Margin status (%)	
Positive	58.82
Negative	41.17
Disease-free survival (months)	
Negative margin group	31.2
Positive margin group	22.6

**Table 2:** Expression of MMP-2 and MMP-9 in the main tumor mass and on the resection margin.

	*N*	Average	*P*
MMP-2			
Tumor	95	98.9160	0.0301
Margin	43	109.0237	
Tumor mass, positive surgical margin	43	91.3581	0.1833
Tumor mass, negative surgical margin	52	105.1658	
MMP-9			
Tumor	106	142.1780	0.1214
Margin	45	171.0924	
Tumor mass, positive surgical margin	45	157.2962	0.0121
Tumor mass, negative surgical margin	61	131.0252	

**Table 3:** MMP-2 and MMP-9 expression in terms of total Gleason score and tumor stage and size.

	Average histoscore	*P*
MMP-2 expression in margin		
Gleason <7	98.103	0.7417
Gleason ≥7	127.453	
Stage 2	113.390	0.5867
Stage 3	102.089	
MMP-2 expression in tumor		
Gleason <7	99.121	0.9389
Gleason ≥7	98.146	
Stage 2	94.942	0.1712
Stage 3	110.771	
Size ≤1 cm	114.165	0.5207
Size >1 cm	97.342	
MMP-9 expression in margin		
Gleason <7	173.391	0.7417
Gleason ≥7	166.927	
Stage 2	173.116	0.9093
Stage 3	170.875	
MMP-9 expression in tumor		
Gleason <7	139.472	0.2597
Gleason ≥7	153.812	
Stage 2	137.432	0.1044
Stage 3	155.930	
Size ≤1 cm	95.000	0.0378
Size >1 cm	143.067	

**Table 4:** ROC analysis.

		AUC	95% confidence interval	*P*
MMP-2, margin	All	0.589	0.413 to 0.749	0.3596

MMP-2, tumor mass	All	0.524	0.408 to 0.638	0.7610
	Negative margin	0.525	0.352 to 0.694	0.8108
	Positive margin	0.693	0.532 to 0.826	0.0988

MMP-9, margin	All	0.551	0.379 to 0.715	0.6352

MMP-9, tumor mass	All	0.626	0.515 to 0.728	0.0582
	Negative margin	0.513	0.343 to 0.680	0.8970
	Positive margin	0.714	0.567 to 0.834	0.0274

**Table 5:** MMP-2 and MMP-9 expression and recurrence regarding cut-off values determined by the Youden index.

		Youden index	95% CI	Associated criterion	Sensitivity	95% CI	Specificity	95% CI
MMP-2	all margins	0.2609	0.1405 to 0.3344	>52.5	100.00	75.3 to 100.0	26.09	10.2 to 48.4

MMP-2 TM	all	0.1679	0.1203 to 0.1839	>158.33	23.81	8.2 to 47.2	92.98	83.0 to 98.1
	neg. margin	0.2475	0.1605 to 0.2910	≤90	76.92	46.2 to 95.0	47.83	26.8 to 69.4
	pos. margin	0.3456	0.1471 to 0.4632	>90	87.50	47.3 to 99.7	47.06	29.8 to 64.9

MMP-9	all margins	0.1827	0.1314 to 0.189	≤115	30.77	9.1 to 61.4	87.50	67.6 to 97.3

MMP-9 TM	all	0.2553	0.1242 to 0.3941	>141.67	65.22	42.7 to 83.6	60.32	47.2 to 72.4
	neg. margin	0.1731	0.1314 to 0.1795	>195	7.69	0.2 to 36.0	75.00	53.3 to 90.2
	pos. margin	0.3974	0.1729 to 0.5718	>175	50.00	18.7 to 81.3	89.74	75.8 to 97.1

CI: confidence interval; TM: tumor mass.

**Table 6:** MMP-2 and MMP-9 expression in terms of cut-off value in tumors with negative margins.

Tumors with negative margins		Recurrence		
		*N *	Yes	No
MMP-9, tumor margin	0 (≤175)	35 (90%)	5 (10%)	39
	1 (>175)	4 (44%)	5 (56%)	9
*P*		0.0065		

MMP-2, tumor margin	0 (≤90)	16 (94%)	1 (6%)	17
	1 (>90)	18 (72%)	7 (28%)	25
*P*		0.1674		

**Table 7:** Analysis of the significance of MMP-9 expression for predicting biochemical recurrence.

	Univariate analysis			Multivariate analysis		
	OR	95% CI	*P*	OR	95% CI	*P*
MMP-2, TM	6.2222	0.6888 to 56.2057	0.1035	5.1721	0.4924 to 54.3318	0.1708
MMP-9, TM	8.7500	1.7411 to 43.9746	0.0078	10.2909	1.6284 to 65.0367	0.0132
				*P* = 0.0052		

OR: odds ratio; CI: confidence interval; and TM: tumor mass.
